# A treadmill running research protocol to assess dynamic visual acuity and balance for athletes with and without recent concussion history

**DOI:** 10.1186/s13102-024-00900-x

**Published:** 2024-05-17

**Authors:** Katelyn M. Mitchell, Kristine N. Dalton, Michael E. Cinelli

**Affiliations:** 1 University of Toronto, Toronto, ON Canada; 2 University of Waterloo, Waterloo, ON Canada; 3 Wilfrid Laurier University, 75 University Ave. W., Waterloo, ON N2L 3C5 Canada

**Keywords:** Vision, Balance, Concussion, Exercise, Cognition

## Abstract

Athletes interpret dynamic visual scenes quickly and accurately during physical exertion. It is important to understand how increased exertion may impact vision and cognition following sport-related concussion (SRC).

**Purpose** To examine the effect of a treadmill running research protocol on the assessment of dynamic visual acuity (DVA) and balance for athletes with and without recent history of SRC.

**Methods** Varsity athletes following recent SRC (CONC=12) were compared to athletes without SRC (ATHLETE=19). The DVA task presented a Tumbling ‘E’ target in four possible orientations during random walk (RW) or horizontal (H) motion at a speed of 30°/s. Participants performed DVA trials standing on a force plate (1000Hz) at four time points: 1) pre-exercise (PRE-EX), 2) immediately (POST1), 3) 10-minutes (POST10), and 4) 20-minutes post- exercise (POST20). Performance was calculated as a change in DVA score from PRE-EX and median response time (RT, ms). Balance control was analyzed using the root mean square of centre of pressure displacement (dCOP).

**Results** Both groups maintained DVA scores for both motion types and exhibited immediate exercise-induced benefits on RT. Both groups had similar change in balance control strategy following treadmill exercise.

**Conclusion** Both groups elicited similar exercise-induced benefits on DVA following exercise. A repeated measures assessment following vigorous exercise may provide meaningful insights about visual and neurocognitive functions for athletes returning to sport following concussion.

**Supplementary Information:**

The online version contains supplementary material available at 10.1186/s13102-024-00900-x.

## Introduction

High performance athletes competing in strategic team sports, such as rugby and ice hockey, process complex, dynamic visual scenes while performing at vigorous levels of physical exertion [1, 2]. Following a sport-related concussion (SRC), athletes may experience a myriad of functional impairments, that commonly affect autonomic regulation, balance control, oculomotor and/or cognitive functions [3–8]. The current return-to-sport (RTS) strategy is progressive in exercise intensity and complexity, focused on restoring an athlete’s capacity for sport performance [9]. Yet, progression through the RTS strategy relies heavily on symptom reporting, with most clinical assessments conducted during resting conditions or in isolation, which may leave meaningful interactions between different clinical domains undetected [10–12]. Previous studies have revealed persisting deficits in dual-tasking, balance control, visual attention, and response times well beyond the recovery of clinical symptoms [13–19]. Such persisting sensorimotor and/or neurocognitive deficits beyond RTS clearance could contribute to an increased risk of subsequent musculoskeletal injury risk up to one year following SRC [20–25]. Therefore, an assessment that integrates multiple clinical domains including vision, cognition, and balance control with vigorous intensity exercise, may better represent the demands of sport to detect persisting impairments following resolution of SRC symptoms [10, 12].

Exercise is a strong mediator of neurocognitive function, such that an acute bout of exercise may lead to immediate improvements in visual attention, response time, and working memory for up to 20-minutes [26–30]. Exercise-induced benefits on cognitive function have been attributed to an increased release of neurotransmitters (e.g., epinephrine) and modulating proteins (e.g., brain-derived neurotrophic factor, BDNF) with physical exertion [30–33]. For instance, BDNF is a regulator of synaptic plasticity and long-term potentiation, specifically in cognitive areas, such as the hippocampus [34–36]. The expression of BDNF may be negatively impacted following traumatic brain injury, such that reduced levels of BDNF have been detected in the hippocampus and pre-frontal cortices of rodents with acute concussion [37]. Implementation of aerobic exercise following concussion appears to be an effective intervention to help restore concentration levels of BDNF in cognitive brain regions [37, 38]. In humans, the physiological effects of concussion injury on BDNF expression remains unclear. However, increased concentration levels of BDNF may help explain why early, structured aerobic exercise (i.e., up to a moderate-intensity of 70% age-predicted maximum heart rate) optimizes time to clinical recovery of SRC symptoms compared to prolonged rest [39–41].

Although there are numerous benefits of moderate aerobic exercise on cognition and symptom recovery, vigorous intensity exercise may provoke a temporary increase in concussion-like symptoms for individuals without recent injury [42–44]. Immediately following vigorous-intensity treadmill exercise, recreationally active young adults have reported an increase in total number and severity of concussion-like symptoms (somatic and cognitive), as well as heightened visual motion sensitivity [42]. However, the provocation of concussion-like symptoms appear to be transient as demonstrated by a significant reduction with improved symptom severity by 20-minutes post-exercise compared to pre-exercise levels [42]. Improved ratings of concussion-like symptoms by 20-minutes post-exercise compared to rest suggests that vigorous intensity exercise may provide delayed benefits beyond acute post-exercise responses. Since team sport athletes perform at higher levels of physical exertion during competition, it is important to understand how cognitive and visual processing may be affected following a bout of vigorous intensity exercise for return to performance following concussion.

The integration of physical exertion with clinical outcome measures may better challenge the limits of neural capacity for athletes with recent SRC to characterize readiness for sport performance demands. In fact, post-exercise exertion testing may be sensitive to neurocognitive impairments in athletes with recent SRC that may not be detected during resting conditions. For instance, a recent study revealed that a portion of athletes who reported a recent SRC history performed worse on a visual executive function task while seated following vigorous aerobic exercise compared to athletes without recent SRC [17]. Based on the constantly-evolving visual processing demands of strategic team sports, it is important to further examine the effects of exercise on more dynamic tasks, such as dynamic visual acuity (DVA). While seated, athletes demonstrate superior DVA performance compared to non-athletes with faster moving targets [45]. Therefore, having athletes perform a DVA task during a more sport relevant posture (i.e., upright standing) and following physical exertion (i.e., vigorous intensity exercise) may be sensitive to detect higher-level visual and neurocognitive impairments for athletes with recent SRC history beyond who are symptom-free.

The purpose of the current study was to examine how a graded, sub-maximal treadmill running protocol affected DVA performance and balance control in athletes with and without recent SRC history. It was hypothesized that athletes with a recent SRC history would perform worse on the DVA task with a positive (worse) change in DVA score and slower RT following exercise throughout the recovery period compared to athlete controls. In addition, it is hypothesized that athletes with SRC would exhibit prolonged postural instability during recovery following vigorous-intensity treadmill running exercise compared to athletes without recent SRC.

## Methods

### Participants

The current study protocol was approved by the Research Ethics Board at the university. All participants provided informed written consent prior to participation in the study protocol. Varsity team athletes (*N* = 31) between the ages of 18 to 25 years-old who identified as male or female were recruited for this study. All athletes played strategic team sports, including ice hockey, rugby, field lacrosse, American football, and soccer (see Table 1). Athletes with recent SRC history (from three months up to approximately three years) who reported no symptoms (CONC =12, female = 6) were compared to athletes who did not report recent incidence of SRC within the previous four years (ATHLETE = 19, female = 12). Exclusion criteria for this study included individuals who report a history of acute concussion less than three months and/or currently symptomatic from recent concussion, vestibular-ocular, neurological, and/or binocular vision disorders (e.g., strabismus, amblyopia, nystagmus). Athlete demographics and SRC injury history are reported in Table 1.

**Table 1:**
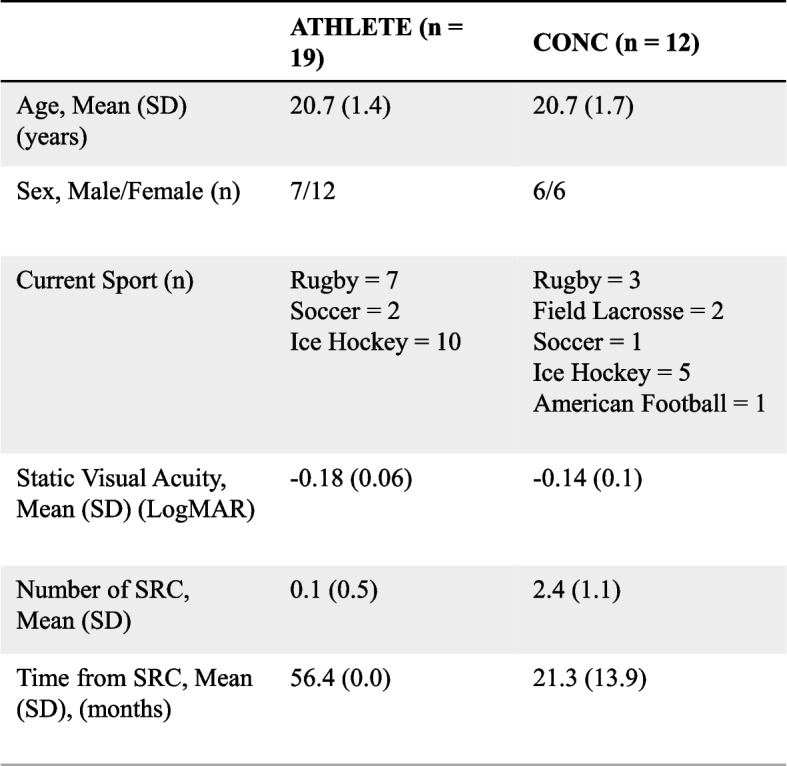
Participant demographic information for each group including age, sex, current sport, visual acuity, and SRC history

### Experimental design

#### Static visual acuity and vestibular-ocular motor assessment

Health and sport history information, including orthopaedic and neurological history, was obtained from a detailed questionnaire that athletes completed prior to participating in the study (see Supplementary material for a copy of the questionnaire). Participants were advised not to engage in exercise for two hours prior and to avoid caffeine for a minimum of three hours prior to the study as it may influence DVA [46]. A Registered Physiotherapist (i.e., primary researcher) assessed each athlete using the Vestibular Ocular Motor Screen (VOMS) and static visual acuity (SVA). The VOMS is a vestibular-ocular motor assessment battery consisting of seven items including smooth pursuits, saccades, near-point convergence, vestibular-ocular reflex (VOR), and visual motion sensitivity. Participants reported if they experienced symptoms of headache, dizziness, and fogginess on an 11-point Likert scale (0 to 10). A positive test is indicated by subjective report of symptoms at an intensity of 2/10 or more on any of the test items [47, 48].

**Fig. 1:**
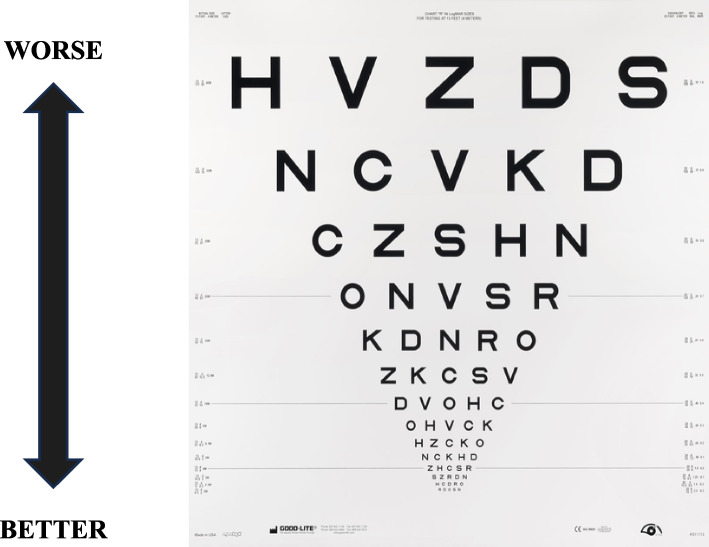
The Early Treatment Diabetic Retinopathy Study (ETDRS) visual acuity charts. Each line represents a change in character size by 0.1 logMAR (log of the minimum angle of resolution)

Both monocular and binocular SVA were assessed using Early Treatment Diabetic Retinopathy Study (ETDRS, Precision Vision; see Fig. 1) vision charts, recorded as the log of the minimum angle of resolution (logMAR). The ability to read a lower line with smaller characters equals a lower (more negative) logMAR and better SVA [49, 50]. Participants were included in the study if they had a SVA score less than or equivalent to 0.3 logMAR (20/40) and a maximum monocular SVA difference of 0.1 logMAR between eyes [51, 52].

#### DVA Assessment

The DVA software program (moV&, V&MP Vision Suite) was developed at the School of Optometry and Vision Science at the University of Waterloo (Waterloo, Canada). A Tumbling “E” target was presented on a 55” monitor in two motion types: random walk (RW) and horizontal (H), at a 4m distance from the viewer. The target motion types exhibit different properties such that RW-motion is continuous and unpredictable, and H-motion that is not continuous and is predictable following a linear path trajectory. For each DVA trial, the target moved at a constant speed threshold of 2.31m/s (30º/sec) [45]. Each participant began the task at a minimum size threshold of 0.5 logMAR above their binocular SVA logMAR score. Participants were instructed to identify the orientation of the Tumbling “E” target (up/down/left/right) quickly and accurately, by selecting the corresponding arrow key on a handheld keypad. Following their response, the target continued moving and changed into a different orientation. If the participants correctly identified the orientation of the “E” in three out of five possible presentations, the target reduced in size by 0.1 logMAR until the participants were no longer able to correctly identify its orientation on at least three out of five presentations. The DVA score was calculated using per-letter scoring, including the smallest target size reached (logMAR) and cumulative errors across the trial (see equation below).

#### Experimental protocol

Prior to beginning the protocol, all participants were familiarized with a practice trial of the DVA task while seated. Each participant began the assessment protocol by completing one DVA trial for RW- and H-motion types in seated that were counterbalanced between participants. During the standing conditions, participants stood with feet together in a narrow Romberg stance on a Bertec® force plate (Columbus, Ohio). Participants first completed a static gaze target trial while fixating their gaze on a “X” target with the monitor positioned at eye level for 45-seconds, followed by one standing trial of each DVA motion type.

The standing conditions at rest were following by the treadmill exercise protocol. Participants wore a Bluetooth chest strap heart rate monitor (Dash Wearables) paired with the Polar Beat mobile application (Polar Electro, Finland). To begin, participants performed a five-minute warm-up, then self-selected a running speed between 5.0 to 7.0 miles per hour (mph) at a 0% incline. Participants then completed 2- to 3-minute treadmill running intervals at their chosen speed that increased incline by 2% each interval. Interval progression was determined by achieving steady state heart rate, such that heart rate in the final minute of the interval was within six beats per minute (bpm) of the previous minute. In addition to heart rate, rating of perceived exertion (RPE) using the Borg Category Ratio 10 scale (CR10; 0 to 10) was recorded at the end of each minute throughout the treadmill protocol. The Borg CR10 scale has been validated and is suggested to be more effective than the traditional Borg RPE scale (6 to 20) [53–57]. The treadmill protocol continued until athletes reached approximately 85% of their age-predicted maximum heart rate (85% HRmax; 220 - age) and/or reported a RPE of 8/10. Participants finished the protocol with a 3-minute walking cooldown and transitioned from the treadmill back to standing on the force platform after removing their footwear.

**Fig. 2:**
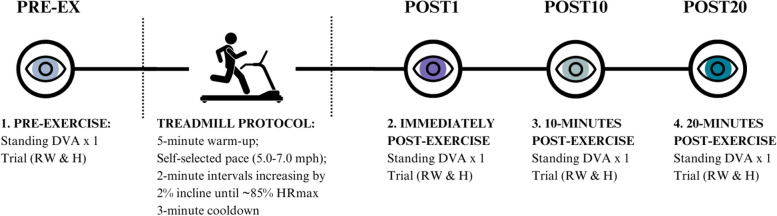
Diagram of the experimental protocol. The DVA task was performed for each motion condition (RW and H) while standing at four time conditions: pre-exercise (PRE-EX) then immediately, (POST1), 10-minutes (POST10), and 20-minutes (POST20) following completion of a graded sub-maximal treadmill running protocol

DVA trials for each motion type were then completed while standing at three time points during following exercise: immediately (POST1), 10-minutes (POST10), and 20-minutes post-exercise (POST20). During the post-exercise recovery period, participants rested while seated between trials without distractions or access to mobile devices. In total, each participant completed one DVA trial while seated and four DVA trials while standing for each motion type (RW- and H-motion), for a total of ten DVA trials (see Fig. 2).

#### Data analysis

Performance on the DVA task was evaluated through scores measured in logMAR. To examine the effects of the vigorous treadmill exercise on DVA, a difference of post-exercise (POST1, POST10, and POST20) from pre-exercise logMAR scores (PRE-EX) were calculated. A lower (more negative) change in logMAR score indicated a better DVA performance and relatively better dual-task performance following exercise. Clinically meaningful difference for DVA (either positive or negative) were identified by a change in DVA score of 0.1 logMAR or greater [58].

DVA logMAR score (per letter scoring) was calculated using the following equation,

LogMAR Score= lowest line read – (-0.02) x number of total errors

Where *lowest line read* represents the smallest target size threshold achieved in a trial. Each character is weighted as 0.02 logMAR, with a total of five targets for a sum of 0.1 logMAR for each size threshold. *Number of total errors* included all incorrect target orientations selected cumulatively for each trial.

Ground reaction forces were collected using a force platform with a sampling frequency of 1000Hz during standing trials (Bertec®, Columbus, Ohio). All balance data was analyzed using a custom MATLAB code (MathWorks, R2023a, Natick, MA). For centre of pressure (COP) analysis, the Root Mean Square (RMS) of COP displacement (dCOP) in the anteroposterior (A/P) and mediolateral (M/L) directions was calculated using the following equation: RMS =$\sqrt{\frac{1}{n} \sum_{i}{x}_{i}^{2}}$

Response time (RT) was measured in milliseconds (ms) from the time a new target orientation was presented until participants selected a response by pressing a button on the handheld keypad. Median RT was normalized to a relative threshold for each participant of 0.5 logMAR above their individual binocular SVA logMAR score. For participants who did not obtain suprathreshold DVA scores within a trial, a minimum of ten responses (0.2 logMAR) above their final response were included.

### Statistical analysis

Participant demographics including medical, sport participation, and concussion injury history for each group were reported as descriptive statistics (see Table 1). All statistical analyses were conducted using *Jamovi* (Version 2.3, 2022) with statistical significance set to an alpha level of 0.05. All data was tested for normality using the Shapiro-Wilk test and homogeneity of variances test (Levene’s). To examine group differences during pre-exercise resting conditions, independent samples t-tests were conducted for binocular SVA, DVA (seated and standing), , and balance control (A/P and M/L dCOP). Effect sizes were calculated using Cohen’s *d* with meaning effects characterized as medium (*d* = 0.5) or large (*d* = 0.8) effects. If the assumption of normality was not met, a Mann-Whitney U non-parametric test was conducted to examine group differences.

A mixed effects linear regression model (MER) was conducted for each dependent variable with fixed effects of group (ATHLETE and CONC) and time condition (PRE-EX, POST1, POST10, and POST20) and with random effects of participant. A Shapiro-Wilk test was conducted to assess the normality of residuals for each model. If the assumption of normality of residuals was not met (*p* < .05), the distribution was examined carefully for outliers (e.g., greater than three standard deviations from the mean). Since MER models may be robust to failure to meet the assumption of normality [59], only the data that fell three standard deviations outside of the mean were identified as significant outliers and removed from the dataset. Dependent variables included difference from resting DVA score, balance control (dCOP in A/P and M/L directions), and median RT for the two motion types. Post-hoc pairwise comparisons using a Bonferroni correction were conducted to interpret significant interactions and main effects of group and time condition. All MER results are presented as estimated means and 95% confidence intervals (95% CI).

## Results

No differences in binocular SVA logMAR scores were evident between groups while seated (t _(29)_ = -0.36, *p* = 0.081, d = -0.67; see Table 1). The VOMS assessment identified one athlete in the CONC group who reported transient 2/10 dizziness with visual motion sensitivity testing. The same athlete did not report any symptoms during the DVA task or treadmill protocol. During the final stage of the treadmill running protocol, all athletes reached the target of 85% age-predicted maximum heart rate (%HRmax) and/or RPE of 8/10 (Mean (SD)= 176.6 bpm (9.8)) and/or RPE (Mean (SD) = 7.8 (1.1)). Immediately after exercise (POST1), CONC had a higher HR (Mean (95% CI) = 122 bpm (116, 129)) compared to the ATHLETE group (Mean (95% CI) = 111bpm (104, 118); t_(29)_ = 2.24, *p* < .05, *d* = 0.83).

### Pre-exercise DVA score

While seated, no differences in DVA logMAR scores were revealed between groups for RW- (Mean (SD): ATHLETE = 0.13 (0.12), CONC = 0.13 (-0.12); U = 111.0, *p* = 0.919) or H-motion conditions (Mean (SD): ATHLETE = 0.14 (0.12), CONC = 0.17 (0.14); t _(29)_ = -0.73, *p* = 0.474). During the PRE-EX condition, there was no significant differences in DVA scores for RW-motion while standing (U = 98.5 *p* = 0.541; see Table 2). Similarly, there were no significant differences revealed between groups for DVA logMAR Scores during PRE-EX for H-motion conditions (t _(29)_ = -1.49, *p* = 0.145; see Table 2).

**Table 2:**
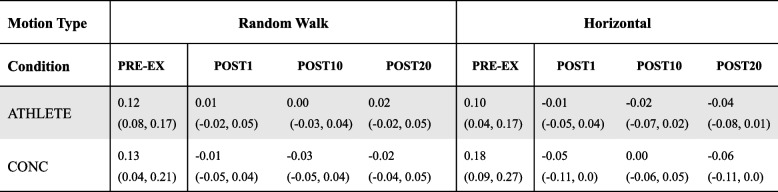
DVA scores (logMAR, Mean (95%CI)) while standing for pre-exercise and relative difference from pre-exercise DVA scores (logMAR, Mean(SD)) between ATHLETE AND CONC following exercise each motion type

### Difference from pre-exercise DVA score:

**Fig. 3:**
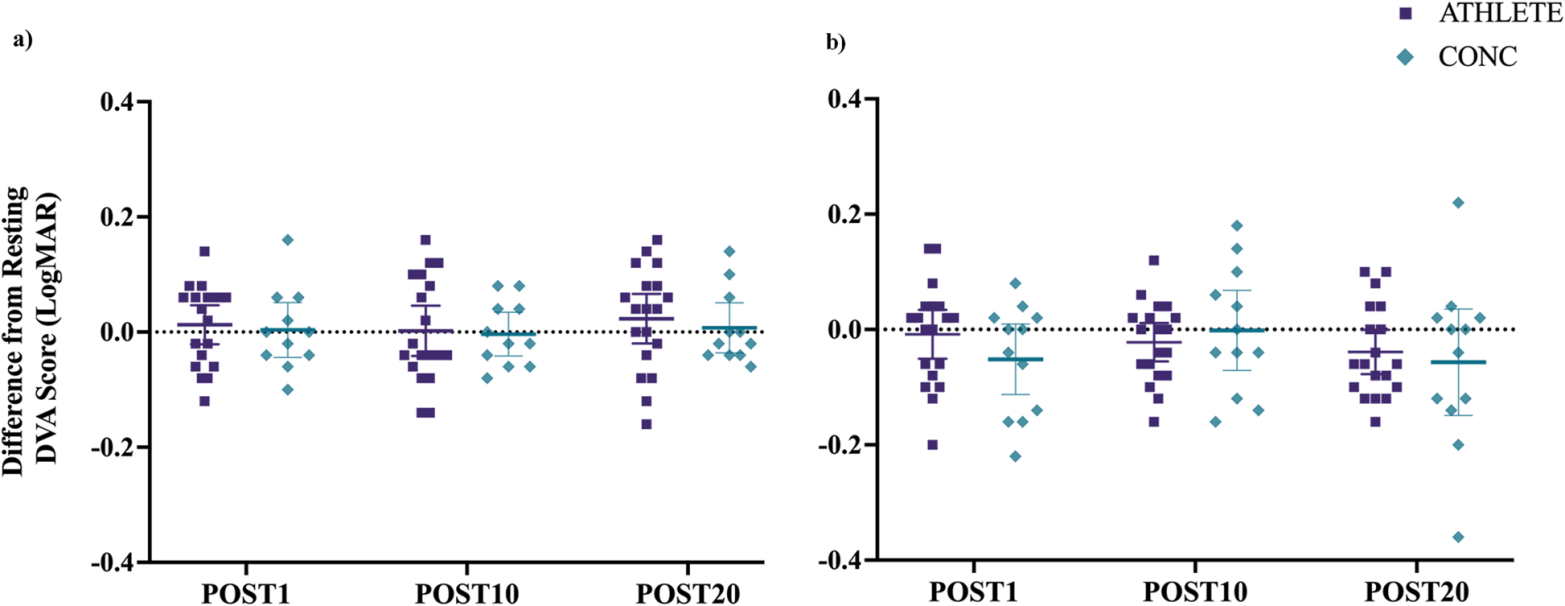
Change in DVA scores for post-exercise time points from pre-exercise plotted as means and 95% CI. Both ATHLETE and CONC maintained performance on the DVA task after completing the treadmill exercise protocol for **a**) RW-motion (*p*=0.371) and **b**) H-motion (*p*=0.628)

For RW-motion, there were no interactions between group and time (F_(2, 58)_ = 0.23, *p* = 0.795) or main effect between groups (F_(1, 29)_ = 0.83, *p* = 0.371; see Fig. 3a) for change in DVA from PRE-EX. For the H-motion condition, there were no interaction effects between group and time (F_(2, 58)_ = 1.26, *p* = 0.292) or main effects between groups (F_(1, 29)_ = 0.24, *p* = 0.628; see Fig. 3b). Percentage of participants with significant clinical changes in DVA score from PRE-EX greater than 0.1 logMAR are reported in Table 3.

**Table 3:**
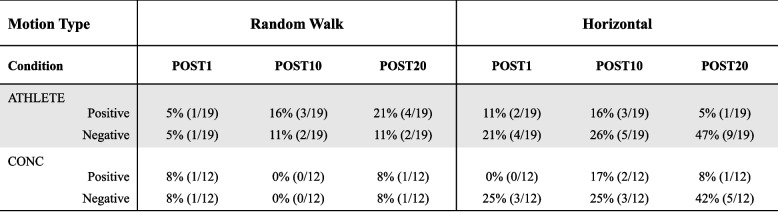
Clinically meaningful change in DVA score >0.1logMAR from PRE-EX indicated by a positive (worse) or negative (better) change; %(n)

### Response time

**Fig. 4:**
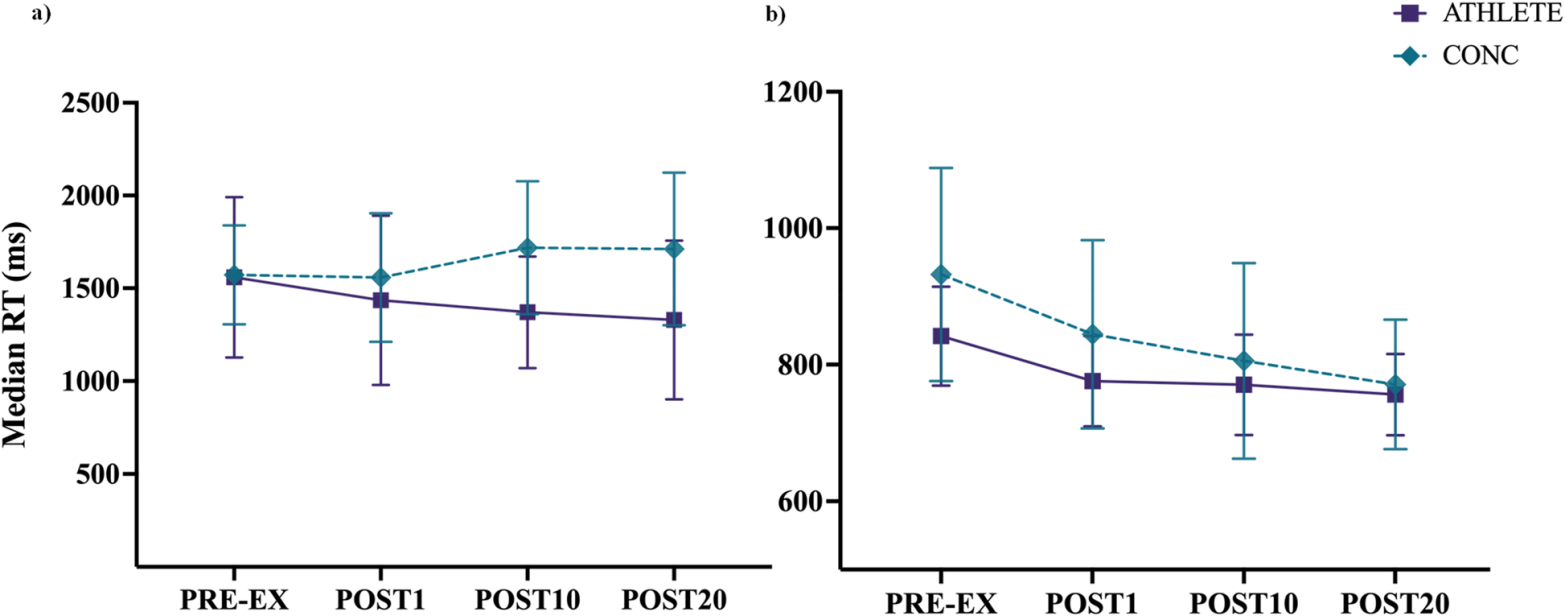
response time (RT; ms) for each time condition from pre-to post-exercise plotted as means and 95% CI for **a**) RW-motion: CONC began to respond more slowly by 10-minutes following exercise compared to ATHLETE; and **b**) H-motion: Both groups improved with faster RT up to 20-minutes after exercise

For RW-motion, one participant in the ATHLETE group was removed as an outlier in the dataset. There were no significant interactions between group and time (F_(3, 84)_ = 1.44, *p* = 0.238). Similarly, no main effects were revealed between group (F_(1, 28)_ = 3.05, *p* = 0.092) or time condition (F_(3, 84)_ = 0.45, *p* = 0.716). Although there is high variability in RT, a portion of the CONC group responded slower at POST10 (Mean (95% CI)= 1718ms (1449, 1987)) and POST20 (Mean (95% CI) = 1712ms (1443, 1980) compared to ATHLETE who improved RT at POST 10 (Mean (95% CI) = 1329ms (1151, 1590)) POST20 (Mean (95% CI) = 1329ms (1109, 1548)) (see Fig. 4a and Table 4).

**Table 4:**
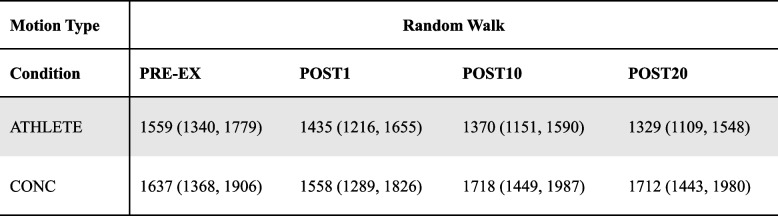
Response time (ms, Mean (95% CI)) during RW-motion DVA measured from the time a new orientation of the DVA target was presented to a response using a keypad for ATHLETE and CONC before and after completing the treadmill running protocol

There was no interaction of condition by group (F_(3, 86)_ = 1.39, *p* = 0.251) or main effect of group (F_(1, 29)_ = 0.73, *p* = 0.401) for the H-motion condition. A significant main effect of time (F_(3, 86)_ = 9.37, *p* < .001) revealed that both groups responded faster following exercise (*p* < .05; see Fig. 4b and Table 5).

**Table 5:**
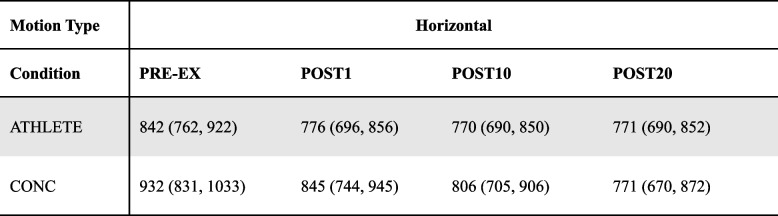
Response time (ms, Mean (95% CI)) during H-motion DVA from the time a new orientation of the DVA target was presented to a response using a keypad for ATHLETE and CONC before and after completing the treadmill running protocol

### Balance control

During the static gaze target trials (ST), both groups maintained balance control similarly for A/P dCOP (t_(27)_ = -0.64, *p* = 0.527) or M/L dCOP (t_(28)_ = -0.65, *p* = 0.519).

### RW-motion

**Fig. 5:**
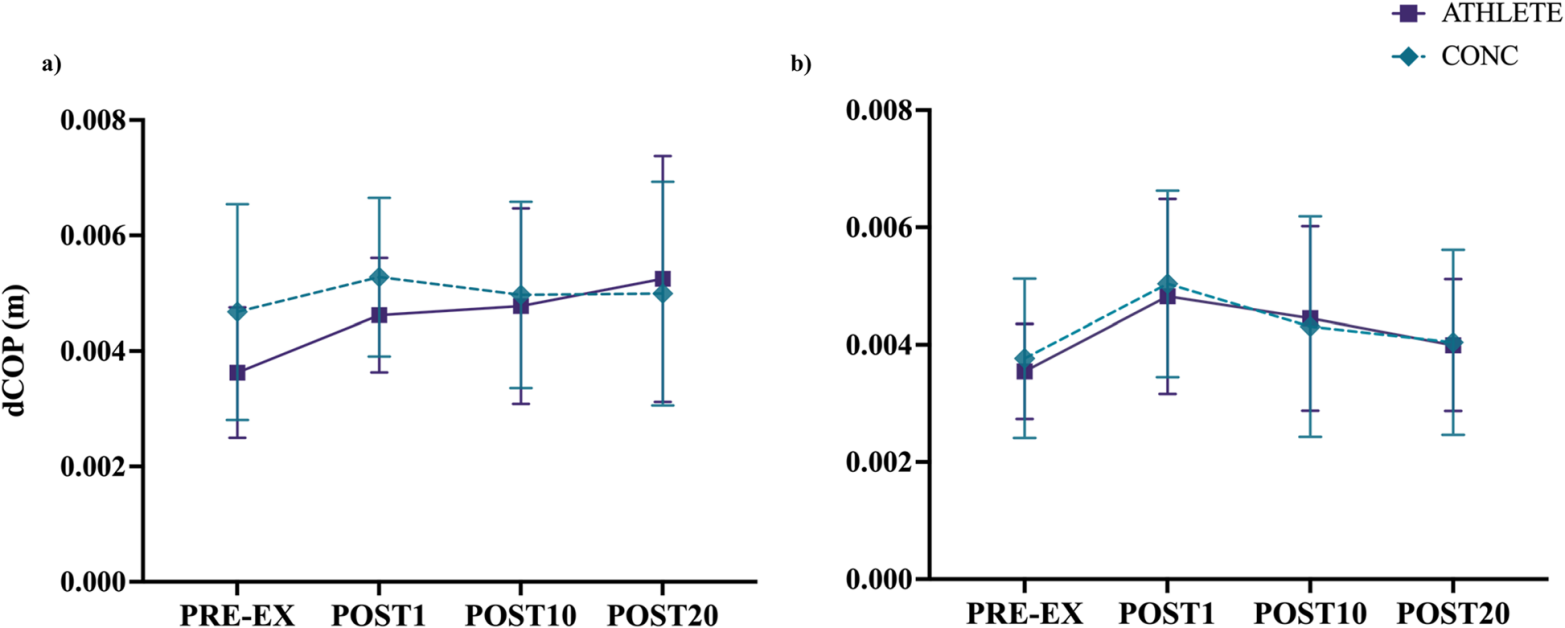
Displacement of COP (dCOP) for RW-motion, plotted as means and 95% CI for all time points, indicating that both groups exhibited a similar change in dCOP immediately following treadmill running exercise in the **a**) A/P direction and in the **b**) M/L direction

For RW-motion, no interactions between group and time (F_(3, 83)_ = 1.66, *p* = 0.183) or main effects were revealed between groups for dCOP in the A/P direction (F_(1, 28)_ = 0.98, *p* = 0.331). There was significant main effect of time for A/P dCOP (F_(3, 83)_ = 4.07, *p* < .05). Post-hoc pairwise comparisons indicated that A/P dCOP increased from at POST1 for both groups (*p* < .05; see Fig. 5a). For M/L dCOP, there were no interactions between group and time condition (F_(3, 85)_ = 0.27, *p* = 0.845) or effects between groups (F_(1, 29)_ = 0.02, *p* = 0.897). A main effect of time was revealed for M/L dCOP (F_(3, 85)_ = 9.92, *p* < .001) with pairwise comparisons indicating that dCOP increased for both groups at POST1 (*p* < .05) (see Fig. 5b).

### H-motion

**Fig. 6:**
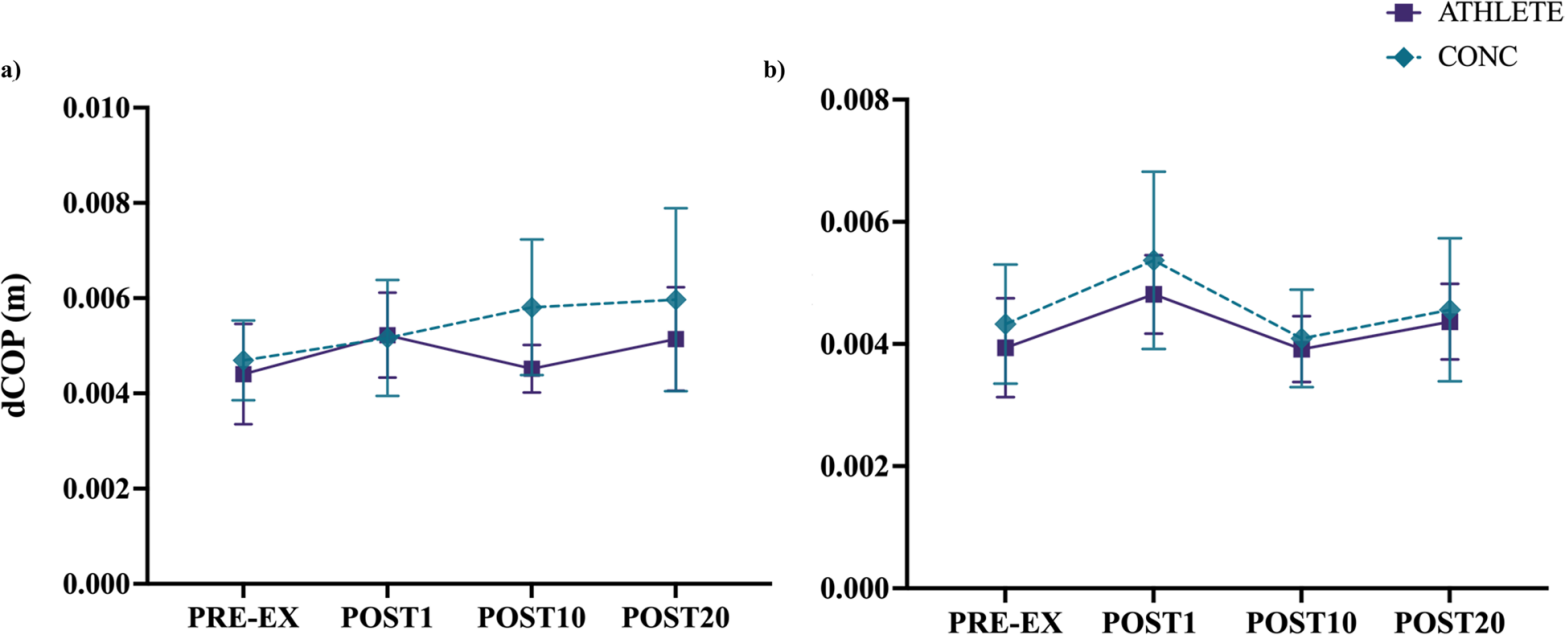
Displacement of COP (dCOP) for the H-motion condition at each time point, plotted as means and 95% CI. Both ATHLETE and CONC exhibited similar balance control changes for dCOP in the **a**) A/P direction and in the **b**) M/L direction anincrease in dCOP immediately post-exercise (*p*<.05)

For H-motion, there were no interaction effects between group and time (F_(3, 83)_ = 0.85, *p* = 0.473). In addition, there were no significant main effects between group (F_(1, 29)_ = 1.75, *p* = 0.197) or time condition (F_(3, 83)_ = 1.63, *p* = 0.190) for A/P dCOP (see Fig. 6a). For dCOP in the M/L direction, there was no interaction between group and time (F_(3, 83)_ = 0.21, *p* = 0.891) as well as no main effect between groups (F_(1, 29)_ = 0.75, *p* = 0.395). A significant main effect of time was revealed for dCOP in the M/L direction (F_(3, 83)_ = 5.71, *p* < .05). Post-hoc pairwise comparisons indicated that M/L dCOP increased during POST1 for both groups (*p* < .05; see Fig. 6b).

## Discussion

The current study sought to examine the effects of a vigorous-intensity treadmill running protocol on DVA performed during dual-task conditions for varsity athletes with (CONC) and without recent SRC history (ATHLETE). It was hypothesized that CONC would exhibit a decline in DVA score and slower RT following treadmill exercise compared to ATHLETE. The primary results of the current study did not support the proposed hypothesis, as both groups maintained or improved relative DVA performance similarly for both motion types (RW- and H-motion) after completing the treadmill running protocol (see Fig. 3).

The current study revealed that vigorous-intensity exercise up to approximately 85% HRmax may benefit performance on a custom DVA task. An increased level of arousal due to physical exertion results in greater activation of neural networks with the release of excitatory chemical mediators [26, 60, 61]. The facilitating effects of vigorous-intensity exercise may act to modulate visuoperceptual processing for athletes that aligns with previous research indicating that athletes may improve visual function following near maximal exertion [62]. Although previous research reported that a greater portion of athletes with previous SRC performed worse on an executive function task while seated following vigorous-intensity exercise [63]. The findings from the current study indicate that both ATHLETE and CONC groups performed similarly with no differences in relative DVA scores immediately following vigorous treadmill exercise (see Table 3).

Similar to DVA scores, the findings from the current study did not support the hypothesis for RT, such that both groups elicited improvements with faster responses on the DVA task immediately following exercise. The current results align with previous studies involving young athletes such that higher intensities of exercise led to a greater influence on the speed of responses, rather than score accuracy [64, 65]. Specifically for H-motion, both groups demonstrated improved RT up to 20-minutes following exercise. The nature of the H-motion condition (i.e., target presented within a brief time window moving along a predictable path), may have induced faster responses as a practice effect due to learning the trajectory of the targets motion. The post-exercise improvements in RT during the RW-motion DVA condition were highly variable for the CONC group, such that a portion of athletes with recent SRC history responded more slowly to maintain relative DVA score accuracy by 10-minutes into the recovery period. Whereas the ATHLETE group continued to improve with faster overall responses during the DVA task up to 20-minutes following treadmill exercise (see Fig. 4a and Table 4). The divergent effect observed for RT at 10- and 20-minutes following an acute bout of exercise resulted in a mean difference of approximately 300ms between groups. The processing time for stationary visual cues is approximately 150ms from the onset of presentation [66], while the latency for long-loop perceptual processing of a stationary visual target has been reported upwards to 400ms [67, 68]. A difference in RT of approximately 300ms to 400ms during a DVA task following exercise, as observed in the current study, requires further investigation to determine if the findings are clinically significant for high performance athletes with recent SRC returning to sport competition. The increased within-group variability observed in both groups indicates that additional factors may also influence vision and cognition following exercise, such as level of fitness, incidence of sub-concussive head impacts for athletes participating in collision sports, and baseline level of cognition and/or attention deficit disorders for athletes [69–72].

Based on the evolution of DVA performance following exercise observed in the current study, in the assessment of vision and neurocognitive performance for athletes may be better detected with repeated measures that compare individual performance over time throughout the post-exercise recovery period. Examining dual-task performance at one discrete time point for athletes post-exercise may overlook important clinical insights highlighting subtle changes in vision and neurocognition that may be apparent following the resolution of concussion-related symptoms. Considering the increased risk of subsequent musculoskeletal injury following RTS, a non-contact ACL tear can occur quite rapidly, within 40ms of ground contact during competition [73, 74]. Such a rapid mechanism of injury cannot be attributable to biomechanical errors, such as dynamic knee valgus, suggesting that visuomotor processing deficits may affect risk of injury. Interestingly, athletes who have sustained non-contact ACL tears have demonstrated neurocognitive impairments similar to those detected following SRC, including slower reaction times and visual processing deficits [75, 76]. The current findings suggest that DVA may be a meaningful clinical measure of both visual function and neurocognition to further explore the boundaries of attentional capacity for athletes using different balance and exercise constraints.

The current findings did not agree with our secondary hypothesis, such that the treadmill running protocol resulted in transient changes in balance control similarly for both ATHLETE and CONC groups. Acute changes in postural stability after vigorous-intensity treadmill running has been attributed to elevated respiration rate, blood flow, muscle fatigue, and altered sensitivity of proprioceptors [77–80]. An increase in ventilation rate post-exercise may bias changes in postural sway along the sagittal plane [80]. Despite the CONC group recording a higher mean HR than ATHLETE immediately following exercise, differences in cardio-autonomic recovery did not appear to impact balance control differently in the A/P direction. For both DVA motion types, athletes in each group demonstrated an acute decrease in postural stability characterized by increased dCOP immediately post-treadmill running that was restored by 20-minutes into recovery similarly for each group in the M/L direction (see Figs. 5 and 6).

There are a number of limitations to consider for the current study. Foremost, the findings may have limited generalizability due to the small sample size of the CONC group with variability in SRC recovery outcomes. This study was planned to begin data collection prior to the COVID-19 pandemic in 2020. Data collection was disrupted by restrictions placed on in-person research and university sport competitions in Canada throughout 2020 and 2021. Due to discontinuation of sport competitions, there was also a reduction in the number of sport-related concussions that occurred during this period of time, which created challenges for recruitment for athletes for the CONC group. In addition, SRC recovery outcomes were based on subjective report by athletes. It is suggested that future studies assess and monitor athletes with SRC from the time of diagnosis throughout the duration of recovery. Further, the varsity athletes included in this study are a niche population that limits generalizability of these findings to the general population. Future research should investigate the relationship between physical exercise and neurocognition after brain injury in more diverse groups. Important personal factors to be considered include age, fitness level, sex, and gender, as well as physical, sensory, and/or cognitive disabilities. More diverse populations may perform differently than experienced athletes, such that the DVA task parameters (e.g., target speed) used in this study may be too challenging for individuals with less athletic experience. In addition, including the analysis of other physiological measures (e.g., gas exchange, blood serum levels) may better characterize post-exercise mechanisms that influence DVA performance and balance control. It is suggested that that continued research should explore how the custom DVA task used in this study compares to other commonly used dynamic visual attention tasks for clinical and performance applications.

## Conclusion

Overall, the results of the current study suggest that a graded treadmill protocol may enhance DVA performance in varsity team athletes with or without recent SRC history, despite the transient effects of running on balance control. Both groups maintained DVA score accuracy and improved RT immediately following treadmill exercise. Improvements in DVA performance suggest that a repeated measures assessment protocol during the post-exercise recovery period may provide meaningful clinical insights to help characterize visual and/or neurocognitive functions for high performance athletes.

### Supplementary Information

Supplementary Material 1. 

## Data Availability

The datasets generated and/or analysed during the current study are not publicly available but are available from the corresponding author on reasonable request.
