# Supplementary material for: A treadmill running research protocol to assess dynamic visual acuity and balance for athletes with and without recent concussion history
Source: BMC Sports Sci Med Rehabil. 2024 May 17;16:112. doi: 10.1186/s13102-024-00900-x (PMC11101338; doi:10.1186/s13102-024-00900-x)
Supplement: Supplementary file 1 — Supplementary Material 1. [file 13102_2024_900_MOESM1_ESM.pdf]

Health Questionnaire

Concussion & Orthopaedic History:

1. Have you ever sustained a concussion injury? Yes / No

    - If yes, how many have been diagnosed?
    - Indicate *the date* of your *most recent* concussion injury.
    - How *much time* did it take to return to sport/achieve full recovery from this injury? (e.x., days, weeks, or months)

2. Please list any *significant lower body injuries or conditions* that may affect/impair your ability to maintain standing balance or walking. (ex: ligament sprains/tears, fractures, muscle strains, chronic ankle instability, leg length discrepancy, arthritic conditions, etc).

    - Indicate *which side* is affected and *what year the injury/condition* occurred.
    - Does this injury affect your ability to stand comfortably, walk, or jog?

3. Please list any other *significant injuries or conditions affecting other regions of the body* including the neck/back and upper extremities (ex: ligament sprains/tears, fractures, muscle strains, arthritic conditions, etc).

    - Indicate *which side* is affected and *what year the injury/condition* occurred.

Health & Vision History:

Age: **Height (ft):** **Weight (lbs):**

1. Have you ever been diagnosed with a neurological conditions or disease? (ex: congenital conditions, traumatic brain injury, tumour, neuromuscular disease, etc)

   Yes / No

   If yes, please indicate the type/name of condition and date of diagnosis.

   Have you ever been diagnosed with a vestibular (ie: inner ear) or visual condition and/or dysfunction? Yes / No

   If yes, please indicate the type of condition and date of diagnosis.

2. Do you have corrected vision? Yes / No

   Please indicate whether you currently wear glasses, contact lenses, etc.

3. Have you been diagnosed with a learning disability and/or attention deficit hyperactvity disorder (i.e. ADHD)? Yes / No

   If yes, please indicate the name of the condition diagnosed.

4. Are you currently taking any prescribed medications? If yes, please list below.

Activity & Sport History:

1. Please list any sports and/or activities that you've participated in competitively in the past for > 1 year in your lifetime.

   - Sport/Years of Participation:

2. Please list any organized and/or recreational sports/activities that you are *currently* participating in.

   - Sport/Years of Participation:

3. Please indicate how many days and hours per week for how many months of the year you participate in the following categories of physical activity and sport-specific training.

   i) Sport-Specific Training (primary sport):

   - Days & hours per week:

   - Months of the year:

ii) Gym- Fitness/Strength Training

- Days & hours per week:

- Months of the year:

iii) Cross Training (other sports/activities)

- Days & hours per week:

- Months of the year:
